# Vestibular rehabilitation's effect over the quality of life of geriatric patients with labyrinth disease

**DOI:** 10.1016/S1808-8694(15)31085-5

**Published:** 2015-10-19

**Authors:** Erika Barioni Mantello, Julio Cesar Moriguti, Antonio Luiz Rodrigues-Júnior, Eduardo Ferrioli

**Affiliations:** 1 Master's degree, doctoral student, Internal Medicine Department, Ribeirão Preto Medical School, São Paulo University. Specialist in Audiology, CFFa. Trained in Audiology, HCFMRP-USP. Speech therapist in the Auditory Health Care Unit, Clinical Hospital, FMRP-USP; 2 Livre-Docente habilitation, Ribeirao Preto Medical School, São Paulo University. Post-doctoral studies, Human Nutrition Research Center on Aging at Tufts University -Boston. Associate professor of the Internal Medicine Department, Ribeirão Preto Medical School, São Paulo University; 3 Doctor in Epidemiology, Adjunct professor of Epidemiology, Ribeirão Preto Medical School, São Paulo University; 4 Post-doctoral studies at Southampton University, England. Doctor in Internal Medicine, Ribeirão Preto Medical School, São Paulo University, Adjunct professor of the Internal Medicine Department, Ribeirão Preto Medical School, São Paulo University. Internal Medicine Department, Ribeirão Preto Medical School - São Paulo University

**Keywords:** metabolic diseases, vascular diseases, elderly, quality of life, vestibular rehabilitation, dizziness

## Abstract

Dizziness is a symptom that affects the population world over, being more prevalent in the elderly due to the process of functional deterioration of the hearing and vestibular systems with aging.

**Aim:**

The objective of this study was to evaluate prospectively the effect of Vestibular Rehabilitation (VR) as treatment for labyrinth disease of vascular and metabolic origin in the quality of life of geriatric patients.

**Methods:**

The study was outlined as clinical-prospective, longitudinal, and observed, with the participation of 40 elder citizens of both genders, divided in 2 groups, dizziness of vascular or metabolic origin. The patients were evaluated and underwent VR - based on Cawthorne and Cooksey's protocol. The statistical analysis from the data was done through the t-Student test, the coefficients of Pearson and Spearman.

**Results:**

based on quality of life scales showed that the individuals treated and assessed improved after Vestibular Rehabilitation.

**Conclusion:**

we concluded that VR, based on the protocols of Cawthorne and Cooksey, could be beneficial to this population

## INTRODUCTION

The elderly population in Brazil is composed of about 15 million people aged 60 years or above (8.6% of the Brazilian populations). In the next 20 years, the Brazilian elderly population may surpass 30 million people and become nearly 13% of the population.^1^ Population aging worldwide is a certain occurrence; in 2025, Brazil will have the sixth largest elderly population in the world.^1^

Aging may be seen as a dynamic and evolving process that includes gradual morphological, functional and biochemical alterations, which becomes more susceptible to intrinsic and extrinsic aggression, ending in death.^2^

The scientific study of aging processes has always occupied a secondary position; there was a tendency not to make time and funding available for studying a life period in which individuals are not economically productive, and may even become dependent on others. More recently, however, the significant increase in the number of elderly people worldwide, and the fact that many of them are still economically active, has generated interest in experimental and clinical studies about various aging-related aspects that until now have been unknown or poorly debated and disseminated.^2^

It is known that the aging process consists of slow and progressive deterioration of organic functions; as the duration of life increases, functional deficiencies become more evident. Dizziness, among them, is considered as one of the most common symptoms in the elderly; it includes various feelings of altered bodily balance, such as vertigo (rotatory dizziness), unbalance, instability, spatial disorientation, a floating feeling, a clouded head feeling and a feeling of being intoxicated.^3^ According to the literature, estimates have suggested that the prevalence of dizziness in the population aged over 65 years will reach 85%; fractures due to falls among this population are responsible for 70% of accidental deaths.^4^

Many factors are responsible for the frequent complaints of dizziness in the elderly. These include decreased spinal mobility, cervical muscle contracture, decreased arterial blood flow, decreased proprioception, auditory, vestibular and visual loss, feeding difficulties, and depression, among others that directly or indirectly affect balance.^5^

The labyrinth, both in its auditory and vestibular portions, is sensitive to disease in other parts of the body. Labyrinthic disease, therefore, may have a vascular, metabolic or hormonal cause, in other words it may be a secondary effect of a systemic organic condition.^6^ Among the most common labyrinthic diseases are Ménière's disease, vascular diseases, metabolic conditions, ototoxicosis, acoustic neurinomas and BPPV. Central vestibular diseases include brainstem and cerebellar conditions, cerebrovascular diseases and diffuse injury to the central nervous system.^7^

An exclusively etiological treatment may not suffice for a favorable outcome in vertigo patients; added benefit may be attained from integrated otoneurological therapy.

This form of treatment includes supervised Vestibular Rehabilitation (VR), the coherent use, if needed, of antivertigo medication for attenuating dizziness and associated symptoms, dietary guidance, and changes in habits that worsen the symptoms. Psychological support may be required for the treatment of depression, anxiety and panic secondary to dizziness.^6^

“VR has been around for a long time; it was first described by Cawthorne in 1944.”^8^ It is a program that includes physical exercises associated with various measures and changes in habits aimed at accelerating vestibular compensation. It has been shown to be an important and effective strategy in the treatment of individuals with body balance disorders, providing significant improvements in the quality of life (QL).

The main aims of VR are: to facilitate visual stabilization and to increase vestibular-visual interaction during head movements; to provide improved static and dynamic stability in situations of sensory conflict; and to reduce individual sensitivity upon moving the head.^9^

Drug and even surgery - albeit rarely - may be used with VR for treating dizziness. Ganança states that to ignore the rational use of antivertigo medication in a multiple therapy approach is a severe mistake, as would also be an error to prioritize medication as the only choice.^10^

Before undergoing surgery, a patient should talk with his or her physician about the probability of a favorable outcome, the nature of potential complications, and decide for prompt surgery or only when the quality of life is deeply affected by vestibular dysfunction. Furthermore, surgery should only be undertaken after having established the disease etiology and after other therapeutic options have been amply used.

The Dizziness Handicap Inventory (DHI) - a test that is easy to apply and to interpret - has been increasingly used in clinical routine and in research, given the significant interference of dizziness in the QL of elderly patients.

Jacobson and Newman (1990), creators of the DHI, developed this test that is composed of 25 questions -based on reports of patients with dizziness - aimed at quantifying behavioral changes resulting from therapy, and gaining useful information for planning the clinical strategy. The questionnaire assesses physical, emotional and functional aspects; scores range from 0 (zero) points for the answer “no,”, 2 points for the answer “sometimes,”, and 4 points for the answer “yes,”.^11^ The difference between pre- and post therapy scores should be at least 18 points for a change to be considered significant in the self-perception of lowered QL resulting from dizziness.^12^^,^^13^

In recent years the DHI has been used for evaluating patient discharge from hospitals, for assessing the effects of medication, of non-medication, of surgery for dizziness, of different therapy in Ménière's disease, the efficacy of laser surgery in BPPV and the efficacy of VR.^11^

Castro (2003), in a thesis defended in 2003,^12^ translated into Brazilian Portuguese and validated the DHI test, which was published in 2004.^14^ Andre applied the DHI test successfully in Brazil in his thesis on the evaluation of elderly patients with BPPV that were treated with VR.^15^

The purpose of this study was to prospectively analyze the effect of VR therapy on the QL of elderly individuals with labyrinthic diseases of vascular and metabolic origin. Specific aims included: obtaining information about the age, sex, main symptoms and the medical diagnosis of the elderly patients; recording the number of therapy sessions between the clinical history and discharge; comparing DHI and QT scale scores before and after VR; verifying possible associations between QT scale and DHI scores before and after VR in the groups with dizziness of vascular and metabolic origin; assessing possible correlations between QT scale scores and the age of elderly patients; and evaluating possible correlations between the number of therapy sessions and the age of elderly patients.

## SERIES AND METHOD

This study was submitted to the Research Ethics Committee of the Ribeirao Preto Medical School Clinical Hospital - São Paulo University (FMRP-USP); authorization was obtained for the study - protocol number 6512/2004. Participants freely agreed to participate and signed a free informed consent form after receiving information about the treatment.

The study was designed as a prospective, longitudinal, observational, treatment-oriented clinical trial including 40 elderly individuals of both sexes, aged between 60 and 84 years. Patients were recruited from the otoneurology outpatient unit, and underwent a clinical otolaryngological evaluation, audiometry and additional tests (laboratory tests, radiology and balance tests) when required for diagnosis. The medical hospital charts of these patients were also consulted for collecting relevant data for the study.

The inclusion criteria were: age over 60 years, an otorhinolaryngological diagnosis of peripheral vestibular dysfunction of vascular or metabolic origin, a history of dizziness, unbalance or falls, and signing the free informed consent form; other diseases, if present, should have been specifically treated or compensated. The exclusion criteria were: associated BPPV (which requires different VR), neurological diseases, neoplasms, diseases of central origin, and patients with severe visual disorders (such as retinal detachment), muscle and skeletal conditions, and psychoemotional disorders that could prevent the exercises from being done adequately.

Forty patients were divided into two groups according to the medical diagnosis; there were 20 patients with labyrinth diseases of vascular origin and 20 patients with labyrinthic disease of metabolic origin.

Patients initially underwent an interview before the research protocol was applied; the aim was to survey the main features of complaints and to set the baseline for therapy. Patients then answered the Brazilian version of the DHI and the QT scale; the latter consisted of patients marking the point which best represented their self-assessment of dizziness in the test date on a 10 cm straight line. The starting point of the line represented no dizziness and the ending point of the line represented maximum dizziness. The line was not marked in any way so that no indication was available which might interfere with the patient's response. At the end of the session, the researcher analyzed the patient's response using a 10 cm ruler to obtain a value for the self-assessment of dizziness from 0 to 10 points at 0.5 cm intervals.

In the first session, all patients received instructions about the treatment, life habits and diet that might favor or interfere with balance, nutrition, and prevention of falls.^16^

After reemphasizing the orientations, therapy was started according to Cawthorne^8^ and Cooksey's protocol^17^ readapted for Brazil by Barbosa et al.^18^ and Pedalini and Bittar.^19^

Treatment basically consists of associated or individual head, eye and trunk movement exercises, and gait with or with no visual, proprioceptive and tactile support, among others. Exercises were demonstrated, explained and trained during sessions in the speech therapy sector. Patients were asked to do the exercises repeatedly two or three times daily at home, depending on each exercise and the treatment stage. Patients were asked to return for evaluation every 15 days. Exercises done wrongly were corrected during the next clinical visit. The protocol we used was chosen for being easier to apply in an elderly population. Patients could be more readily motivated by these easier exercises, which is essential for the progression of therapy.

The exercises were shown through drawings and in clear language, which were provided for the patients (see Figure 1, Figure 2, Figure 3, Figure 4, Figure 5, Figure 6, Figure 7, Figure 8, Figure 9, Figure 10, Figure 11).

**Figure 1:**
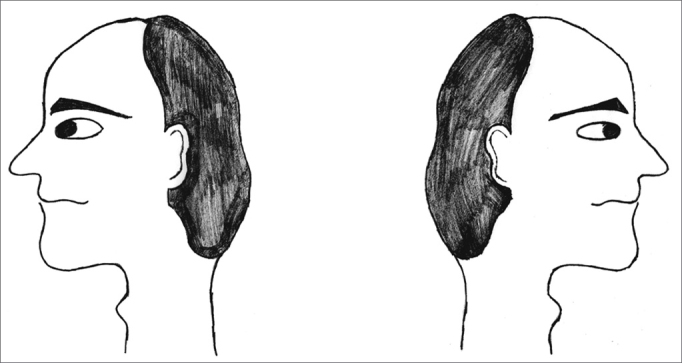
Sideways (right and left) head movement.

**Figure 2:**
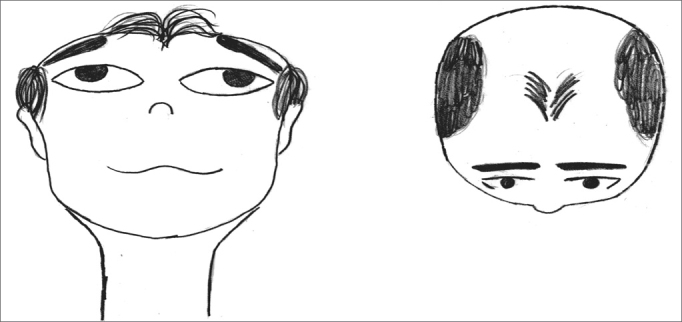
Upward and downward head movement.

**Figure 3:**
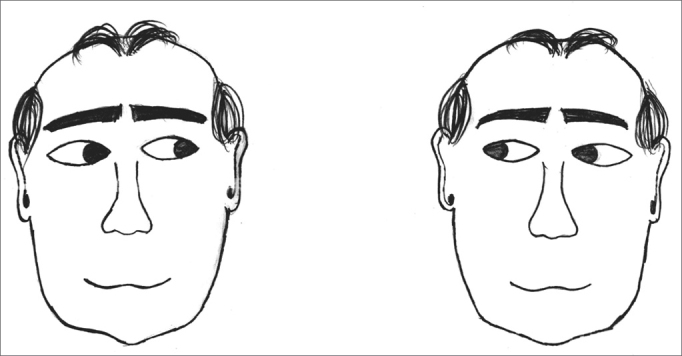
Sideways (right and left) eye movement.

**Figure 4:**
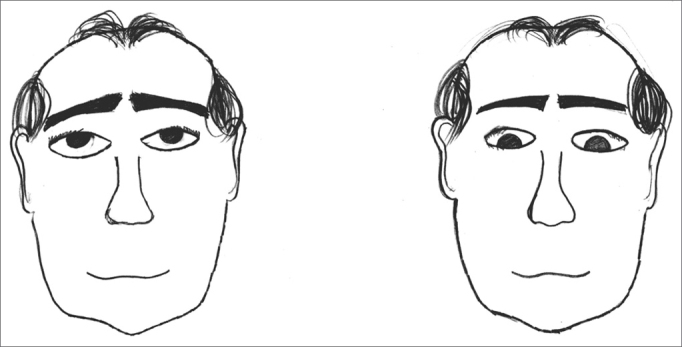
Upward and downward eye movement.

**Figure 5:**
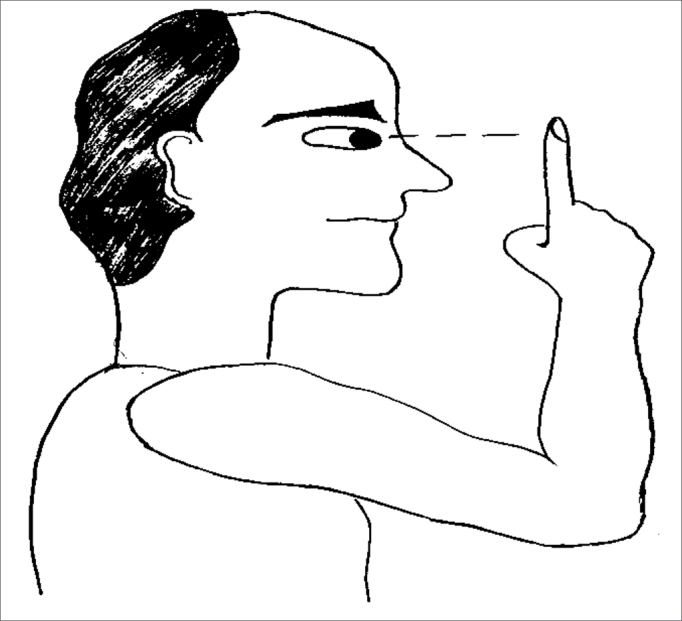
Fixing the gaze on a finger that is moved further and closer.

**Figure 6:**
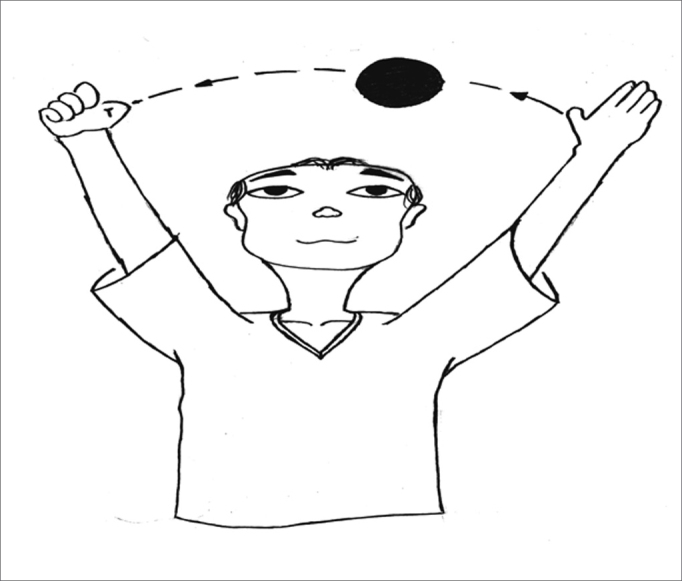
Throwing a ball from one hand to the other while keeping the gaze fixed.

**Figure 7:**
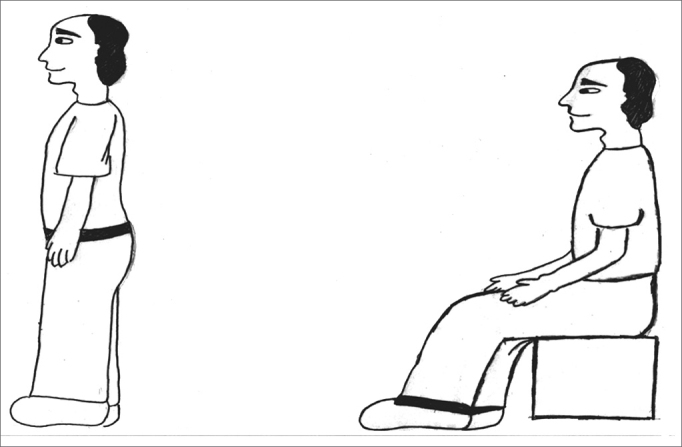
Sitting, standing up and sitting again.

**Figure 8:**
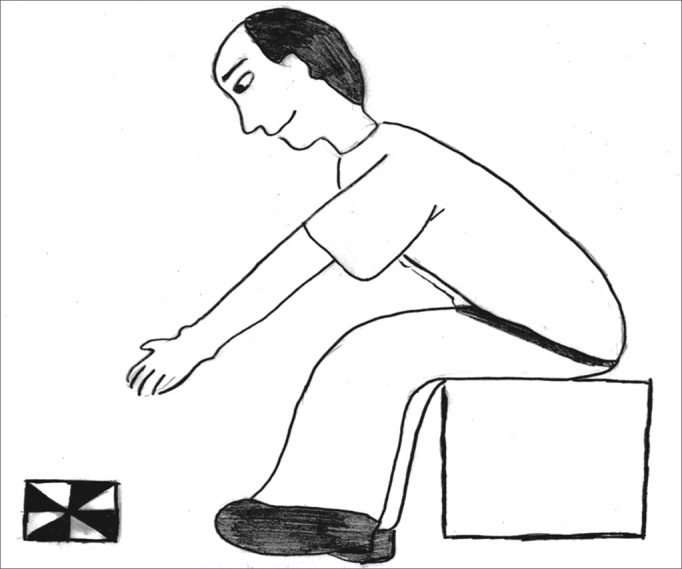
Picking up objects on the floor while keeping the gaze fixed.

**Figure 9:**
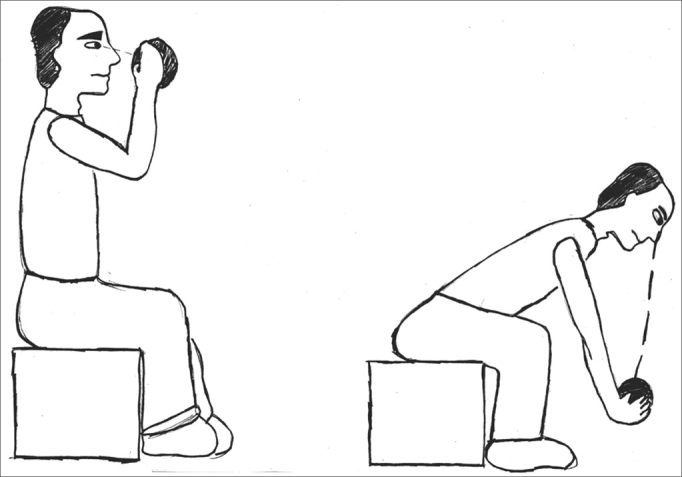
Lifting and putting down a ball while keeping the gaze fixed.

**Figure 10:**
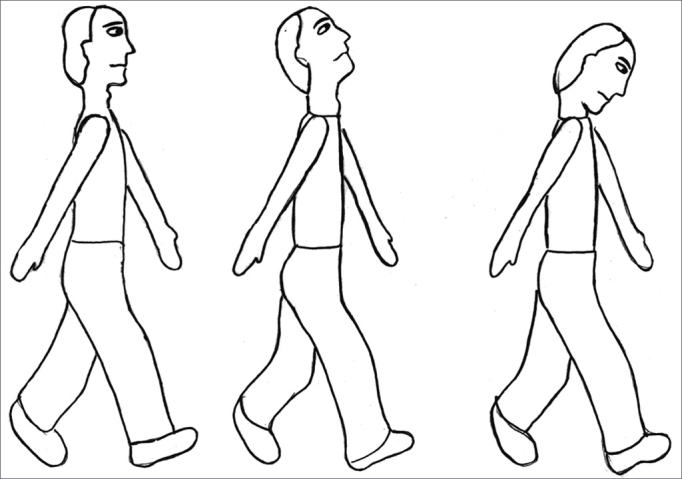
Walking in a straight line while gazing forward; walking in a straight line while looking upwards and downwards.

**Figure 11:**
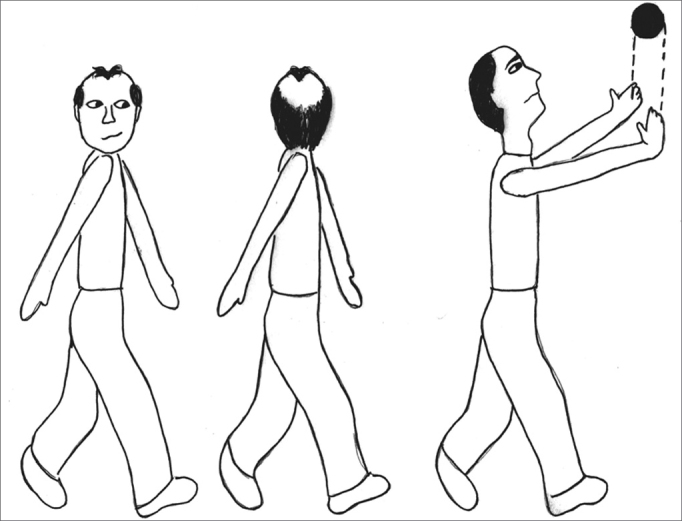
Walking in a straight line while looking sideways; walking in a straight line while throwing a ball from one hand to the other.

At the end of therapy and before discharge, each patient again answered the DHI test and the QT scale to verify their performance before and after treatment. An otorhinolaryngologist evaluated all cases within four months after treatment to decide for discharge or for a new treatment approach - which was discussed with the attending physician - depending on the progression of each case.

Student's t test was used in the statistical analysis for comparing paired samples, based on a bilateral hypothesis test. Pearson's and Spearman's coefficients were used in correlation studies.^20^^,^^21^ Significance was considered as p < 0.05. Student's t test was used for comparing the “metabolic,” and the “vascular,” groups, using the response variables described above; differences between values observed before and after the treatment were taken into account. These comparisons (between pre- and post treatment stages) included the values seen in each group using the paired form of hypothesis testing, as there was no significant difference in the values.

## RESULTS AND DISCUSSION

Females comprised 62.5% of the total samples, 65% of the group with dizziness of metabolic origin (GM) and 60% of the group with dizziness of vascular origin (GV).

In the total sample, 47.5% of the elderly patients were aged between 60- and 69 years, 40% were aged between 70 and 79 years, and 12.5% were aged between 80 and 89 years; the mean age of all patients in this study was 70.2 years. In the literature, similar studies on dizziness reveal mean ages of 70.9 years,^22^ 71.0 years^12^ and 72.2 years.^4^

Altered glucose metabolism is mentioned as the main metabolic change leading to vestibulocochlear disorders, as was seen in the GM.^23^^,^^24^ Features of diabetes mellitus, such as muscle degeneration, decreased proprioception and difficult vestibular compensation explains why this disease is so prevalent in causing vestibular conditions of metabolic origin.^25^

Baloh^26^ has stated that elderly patients with dizziness of vascular origin in general present high systemic arterial blood pressure, followed by heart diseases, which corroborate the findings in the GV.

An elevated incidence of psychological problems was observed; this was also a finding in Monzani et al.'s study.^27^ Ganança has explained that “the coexistence of dizziness, anxiety, depression and fear is common in day-to-day otoneurological practice; this crystallizes the functional interaction between those systems that are responsible for psychic and physical balance.,”^10^

Dietary and life habits were not controlled before the treatment in 60% of patients in the GV and in 55% of patients in the GM; 65% of GV patients and 50% of GM patients were sedentary. After counseling, 90% of the sample started to control the diet and other habits, and over 70% of patients started going for daily or weekly walks. It is important to note that these data were not treated statistically.

Similar to Ganança, Dias and Ganança's^16^ findings, we observed that investing in adequate counseling time for patients with dizziness is essential for compliance with the treatment; this step is as important as VR exercises, making it easier for patients to believe in the treatment, to do the exercises, to accelerate compensation and to maintain the favorable results after discharge. It is known that alcohol, smoking, sugar, salt, saturated fat, caffeine and lack of exercise should be significantly reduced or eliminated from the lives of patients with vertigo, since they may exacerbate cochleovestibular symptoms and slow down vestibular compensation. Such slowness was observed in our study; patients that did not follow the orientation required more treatment sessions, although this slowness was unrelated with a worse response to VR.

Table 1 shows the difference between mean values (and the standard deviation) of the study variables in both groups (vascular and metabolic origin of dizziness). The differences were not significant between both groups.

**Table 1:** Mean differences between the study variables in the metabolic and vascular dizziness groups, and the standard deviation.

Variable	Group	
	Metabolic	Vascular
Quantification of dizziness	−3,6 ± 4,93	−3,0 ± 6,37
Dizziness Handicap Inventory	−47,8 ± 22,91	−46,8 ± 16,84
Physical score	−17,9 ± 7,91	−16,6 ± 4,59
Emotional score	−11,5 ± 9,51	−12,6 ± 9,47
Functional score	−18,4 ± 8,79	−17,6 ± 6,85

**Chart 1:**
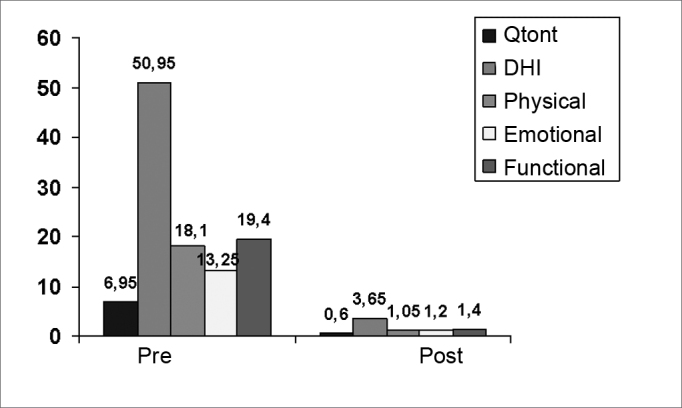
Graphic representation of mean differences between the variables quantification of dizziness (Qtont), the Dizziness Handicap Inventory (DHI), the physical score (físico), the emotional score (emocional), and the functional score (funcional), before and after therapy.

Since the previous results were not significantly different between groups, we analyzed all of the patients regardless of a diagnosis of dizziness of metabolic or vascular origin. Chart 1 shows the difference between means before and after the treatment with VR.

Involvement of sensory systems (vision and proprioception) and of the central nervous system, precarious skeletal, muscular and joint conditions, altered bodily weight and memory loss are some of the factors that, singly or jointly, may be present in the elderly, which compromises VR results.^28^ These factors were exclusion factors in our study, which might explain the favorable results found in both groups after VR.

Findings in the current study about the efficacy of VR in elderly patients are similar to those of Whitaker et al.,^29^ Cohen^30^ and Bittar et al.^31^ Improvement in all DHI scores following VR in elderly patients with improved quality of life was observed in our patients, similar to findings by Castro,^12^ Ganança et al.,^13^ Silveira, Taguchi and Ganança,^32^ and Sznifer et al.^33^ These results suggest that there is no age limit for the VR program, as also stated by Norre and De Weerdt,^34^ and Shepard, Telian and Wheelock.^35^

It is important to assess the loss of QL in patients with vertigo - particularly in elderly patients - to quantify the impact of vertigo on daily life functions and to help chose the evaluation and treatment methods for this condition.

In the current study, the functional score showed the highest pre- and post-treatment differences, and thus the highest impact on the QL, closely followed by the physical score and to a lesser degree, the emotional score. Similar results were found in the papers published by Silveira, Taguchi and Ganança.^32^ The physical score was highest in a study by Ganança et al.^14^ Physical scores in the Brazilian version of the DHI mainly evaluate the onset of dizziness upon changes in head positions and bodily tilting, which are specially frequent in BPPV cases; this diagnosis was excluded in our study, but not in other papers that we consulted in the literature, in which physical scores were higher in elderly patients.

Enloe and Shields^36^ and Roberson and Ireland^37^ found that physical and functional scores were more compromised than emotional scores, as was found in our study.

The number of therapy sessions varied from 4 to 10 sessions in this study. Nishino et al.^38^ used 1 to 15 sessions. Ganança et al.^13^ reported that most of their patients attended up to five treatment sessions. Silveira, Taguchi and Ganança^32^ underlined the need for treatment sessions for vestibular compensation. Different treatment protocols - which may vary depending on exercise difficulty - in various published studies may explain the reported variations in the duration of VR.

We agree with Herdman,^39^ who stated that compensation is a process in which a function adjusts to a change in sensitivity, and that this varies with age; younger individuals accelerate vestibular compensation more easily. Maximum compliance with the treatment is, therefore, needed in elderly patients for attaining similar results. This requires more treatment sessions in elderly patients, compared to young individuals.

There was no significant correlation (Spearman) between the age of patients in the GM (r = 0.352 and p = 0.128) and the GV (r = 0.028 and p = 0.908) and the number of treatment sessions (r = 0.131 and p = 0.419 for the total sample). There was no significant correlation (Spearman) between the dizziness quantification scale and the number of treatment sessions (r = 0.016 and p = 0.887 for the total sample). There was also no significant correlation between the DHI and the number of treatment sessions (r = 0.143 and p = 0.207 for the total sample).

According to the treatment reports, patients that attended more treatment sessions were not the oldest or those with the highest performance differences in pre-and post-treatment evaluations (QT and DHI), but rather, those patients that had more difficulties to understand the instructions, or that had less physical mobility for doing the exercises, or that had psychological problems, and those that did not fully comply with dietary and life change orientations. Bittar et al.^31^ have stated that the cognitive and physical deficiencies of aging, such as difficulties in understanding and in executing the exercises, physical and psychic limitations and lack of motivation, are complicating factors that may delay clinical improvement; this would require more training time for adequate integration into the treatment program. These findings apply to that part of our sample that attended more treatment sessions.

**Chart 2:**
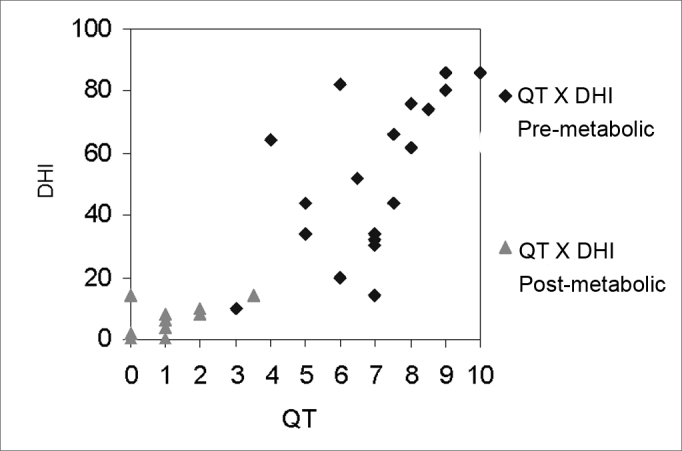
Graphic representation of the correlation between the quantification of dizziness scale and the DHI test, before and after treatment, in the metabolic dizziness group.

**Chart 3:**
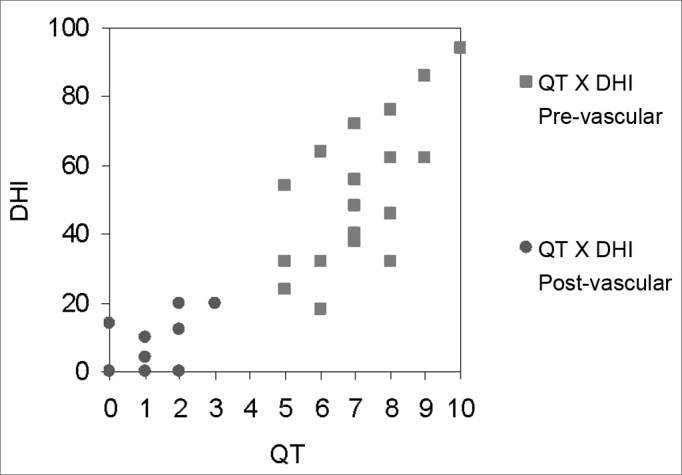
Graphic representation of the correlation between the quantification of dizziness scale and the DHI test, before and after treatment, in the vascular dizziness group.

Chart 2 shows the significant correlation (Pearson) between the QT scale and the DHI test before and after treatment in the GM (r = 0.875 and p < 0.0001); Chart 3 shows the same for the GV (r = 0.915 and p < 0.0001). This value for the full sample was r = 0.894 and p < 0.0001. No paper was found that had applied the QT scale. Knobel et al.^40^ applied this scale for evaluating patients with tinnitus and auditory hypersensitivity, and described good results. This demonstrates that further studies using the QT scale should be undertaken in larger and different populations to increase the scope of these findings. In our paper, we concluded that the QT scale is a simple, easy and rapidly applied test that may be correlated with the DHI, an internationally validated and applied test.

## CONCLUSION

Elderly patients with labyrinthic disease of metabolic or vascular origin were aged between 60 and 69 years, with a mean 70.2 years. In most of these patients, their main symptoms were tinnitus, hearing loss, unbalance and dizziness. The prevalent diagnosis in the metabolic group was diabetes mellitus, and the prevalent diagnosis in the vascular group was systemic high blood pressure.

The duration of treatment by VR was about four therapy sessions, reaching eight sessions. Age was not considered as a limiting factor on the response to therapy.

The conclusion is that VR (based on Cawthorne and Cooksey's protocol) in elderly patients with vascular or metabolic labyrinthic disease was effective in improving the quality of life of these patients. Although there were improvements in both groups following therapy, according to the DHI, there were no differences in the effect of therapy on the quality of life between both groups (metabolic and vascular dizziness). In this study there was a significant correlation between the dizziness quantification scale before and after the treatment, and the DHI before and after treatment.

Taking into account the epidemiological data on aging in Brazil, and knowing that in most of the elderly patients with otoneurological conditions the origin of disease is vascular or metabolic, we conclude that VR may be beneficial in this population. It is important that this physiological form of therapy be disseminated among healthcare professionals working in gerontology teams.
